# Approaches to Evaluating Digital Health Technologies: Scoping Review

**DOI:** 10.2196/50251

**Published:** 2024-08-28

**Authors:** Anneloek Rauwerdink, Pier Spinazze, Harm Gijsbers, Juul Molendijk, Sandra Zwolsman, Marlies P Schijven, Niels H Chavannes, Marise J Kasteleyn

**Affiliations:** 1 Department of Radiology and Nuclear Medicine Amsterdam University Medical Center Amsterdam Netherlands; 2 Department of Public Health and Primary Care Leiden University Medical Centre Leiden Netherlands; 3 Department of Surgery Amsterdam University Medical Center Amsterdam Netherlands; 4 Digital Health Amsterdam Public Health Institute Amsterdam Netherlands; 5 Department of Medical Informatics, eHealth Living & Learning Lab Amsterdam University Medical Center University of Amsterdam Amsterdam Netherlands; 6 Amsterdam University Medical Center Amsterdam Netherlands; 7 Amsterdam Gastroenterology Endocrinology Metabolism Amsterdam Netherlands; 8 National eHealth Living Lab Leiden Netherlands

**Keywords:** ehealth, digital health, telemedicine, methods, evaluation, literature review, DHI, health technology, health technologies, telehealth, e-health, telemedicine, scoping review, digital health intervention

## Abstract

**Background:**

Profound scientific evaluation of novel digital health technologies (DHTs) is key to enhance successful development and implementation. As such, we previously developed the eHealth evaluation cycle. The eHealth evaluation cycle contains 5 consecutive study phases: conceptual, development, feasibility, effectiveness, and implementation.

**Objective:**

The aim of this study is to develop a better understanding of the daily practice of the eHealth evaluation cycle. Therefore, the objectives are to conduct a structured analysis of literature data to analyze the practice of the evaluation study phases and to determine which evaluation approaches are used in which study phase of the eHealth evaluation cycle.

**Methods:**

We conducted a systematic literature search in PubMed including the MeSH term “telemedicine” in combination with a wide variety of evaluation approaches. Original peer-reviewed studies published in the year 2019 (pre-COVID-19 cohort) were included. Nonpatient-focused studies were excluded. Data on the following variables were extracted and systematically analyzed: journal, country, publication date, medical specialty, primary user, functionality, evaluation study phases, and evaluation approach. RStudio software was used to summarize the descriptive data and to perform statistical analyses.

**Results:**

We included 824 studies after 1583 titles and abstracts were screened. The majority of the evaluation studies focused on the effectiveness (impact; 304/824, 36.9%) study phase, whereas uptake (implementation; 70/824, 8.5%) received the least focus. Randomized controlled trials (RCTs; 170/899, 18.9%) were the most commonly used DHT evaluation method. Within the effectiveness (impact) study phase, RCTs were used in one-half of the studies. In the conceptual and planning phases, survey research (27/78, 35%) and interview studies (27/78, 35%) were most frequently used. The United States published the largest amount of DHT evaluation studies (304/824, 36.9%). Psychiatry and mental health (89/840, 10.6%) and cardiology (75/840, 8.9%) had the majority of studies published within the field.

**Conclusions:**

We composed the first comprehensive overview of the actual practice of implementing consecutive DHT evaluation study phases. We found that the study phases of the eHealth evaluation cycle are unequally studied and most attention is paid to the effectiveness study phase. In addition, the majority of the studies used an RCT design. However, in order to successfully develop and implement novel DHTs, stimulating equal evaluation of the sequential study phases of DHTs and selecting the right evaluation approach that fits the iterative nature of technology might be of the utmost importance.

## Introduction

### Background

Health care, traditionally a slow adopter of digital innovation, has recently seen an acceleration in the use of digital tools and systems. A transition to more remote patient care and associated services is urgently needed due to an aging global population [1]. In a highly regulated sector in which data privacy and health outcomes are vital, there needs to be a high level of scrutiny and standardized evaluation frameworks in place to limit the inherent risk in rapid innovation and ensure successful outcomes. With an increasing focus on digital health research, it is important to take stock of digital health evaluation methodologies to further guide prospective studies [2].

The World Health Organization (WHO) plays an important role in guiding and accelerating the development of digital health interventions and health innovation globally. In 2018, the WHO developed a *classification scheme of digital health interventions*, aiming to promote a comprehensive and standardized language for health program planners [3]. The scheme organized digital health interventions into the following primary user groups: clients, health care providers, health systems or resource managers, and data services (Multimedia Appendix 1).

Another valuable framework was developed by the National Institute for Health and Care Excellence (NICE) together with the National Health Service in England in 2019 [4,5]. They created the Evidence Standards Framework (ESF) for digital health technologies (DHTs) in order to promote greater consistency when evaluating or commissioning DHTs and thereby enhance the level of scrutiny, which is generally lower than the level of evidence required for drugs or devices. The subjects of NICE’s ESF are system services (evidence tier 1); inform, simple monitoring, communicate (evidence tier 2); preventative behavior change, self-manage (evidence tier 3a); and treatment, active monitoring, calculate, and diagnose (evidence tier 3b).

**Figure 1:**
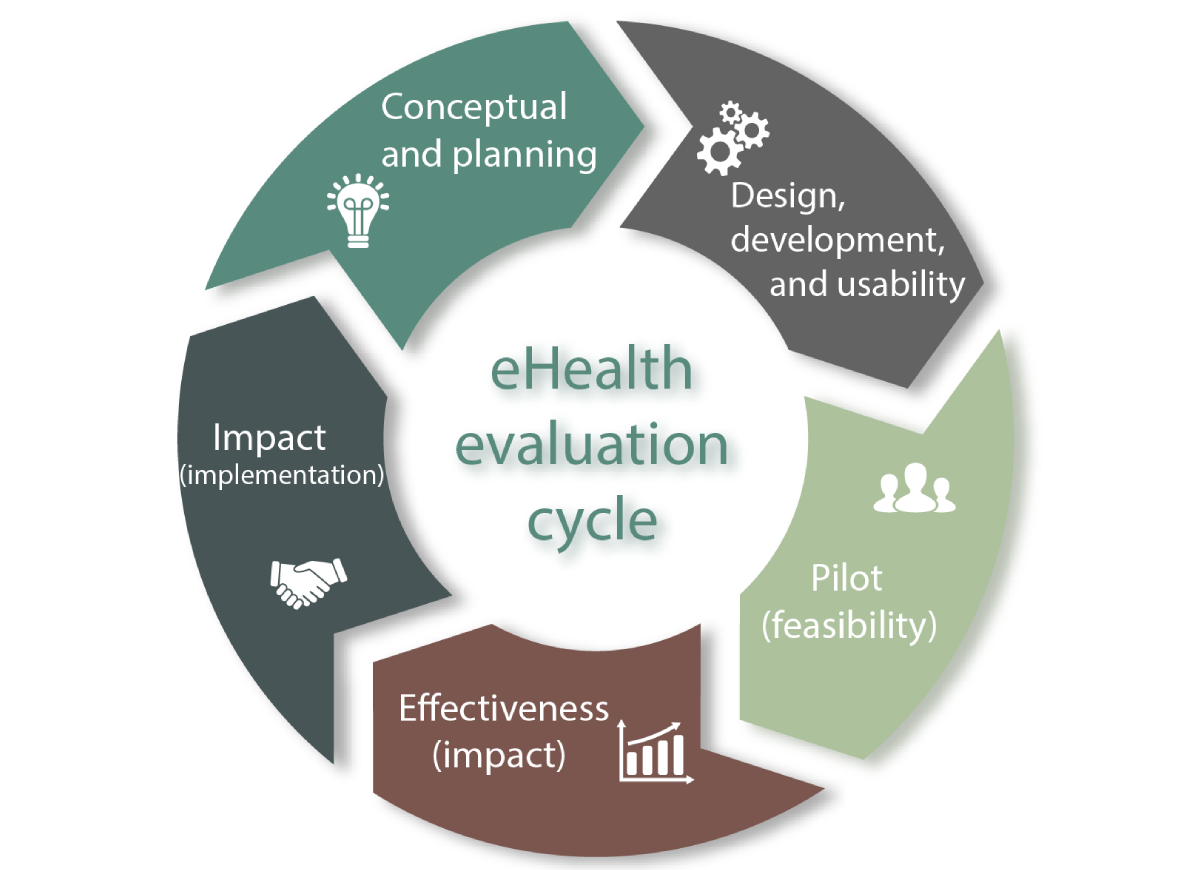
eHealth evaluation cycle.

The WHO’s digital health classification scheme and the NICE’s ESF could work synergistically, providing a much needed standardized and accepted framework for the varied stakeholders involved in digital health to evaluate and improve the development of evidence-based digital health solutions. To provide further granularity to the focus of digital health research, evaluating the sequential study phases—conceptual to implementation—is important [6,7]. Therefore, we previously developed the *eHealth evaluation cycle* (Figure 1) based on existing eHealth evaluation frameworks [8]. The *eHealth evaluation cycle* contains the following 5 consecutive study phases: conceptual and planning; design, development, and usability; pilot (feasibility); effectiveness (impact); and uptake (implementation). In the online version of the *eHealth evaluation cycle*, one can find a synopsis of evaluation approaches (all the methods, study designs, frameworks, and other philosophical approaches) that could be used to evaluate a particular study phase [9]. For example, a “concept mapping study design” can be used to gather information in the conceptual and planning study phase, and an “economic evaluation” can be used in the uptake (implementation) study phase [10]. There are also several types of systematic reviews within the *eHealth evaluation cycle*. For instance, a meta-analysis can be found within the effectiveness (impact) phase, and a narrative review can be found in the conceptual and planning phase when one is, for example, at the start of developing a new DHT and aims to define what is already known.

Although the number of DHT publications has exponentially grown in recent years, not much is known about the use in daily practice of the consecutive study phases [11]. There are indications that there is too much focus on evaluating *effectiveness* instead of, for example, usability testing [12]. In addition, large-scale implementation of DHT often fails [13-15]. Therefore, in order to potentially improve DHT evaluation and therefore implementation of DHTs, the following research question will be addressed in this study: What is the actual practice of the consecutive evaluation study phases described in the literature?

### Aim and Objective

The aim of this study was to develop a better understanding of the daily practice of the *eHealth evaluation cycle*. Therefore, the research objectives were to (1) conduct a structured analysis of data from the literature to analyze the practice of the evaluation study phases and (2) determine which evaluation approaches are used in which study phase of the eHealth evaluation cycle.

## Methods

### Overall Design

We performed a scoping review subdivided into 2 phases: (1) systematic literature search to find articles concerning the evaluation of DHT, followed by the extraction of data from the selected articles; (2) performing a structured data analysis. We followed the PRISMA (Preferred Reporting Items for Systematic Reviews and Meta-Analyses) guidelines (Multimedia Appendix 2).

### Search Strategy

The online PubMed database was systematically searched using the MeSH term “telemedicine” in combination with a wide variety of “evaluation approaches” (see Multimedia Appendix 3 for the complete search string). Articles written in English published in the year 2019 were included. The year 2019 was chosen since it is the most recent “pre-COVID-19” year cohort. Herewith, we aimed to avoid possible skewing of results by the temporary surge in COVID-19–related DHTs.

### Inclusion and Exclusion Criteria

The following inclusion and exclusion criteria were used. The inclusion criteria consisted of any original, peer-reviewed study evaluating a specific DHT in one or multiple study phases of the eHealth evaluation cycle; all types of literature reviews describing a specific DHT; and tele-education studies when closely related to the outcomes of health care.

The exclusion criteria were papers not written in English; studies not related to DHTs; nonpatient-focused studies (eg, evaluation of technology alone [ie, sensitivity of sensors]); and poster presentations, protocol studies, and opinion papers.

### Study Selection

Articles were included based on screening the title and abstract by 2 pairs of researchers. HG and PS reviewed articles with titles starting with the letters A through K, and AR and JM reviewed articles with titles starting with the letters L through Z. Two test series of 20 cases were discussed between the 4 researchers to reach a satisfactory level of consensus before the pairs began screening the titles and abstracts. Rayyan QCRI online software was used to collectively screen articles [16].

Once the inclusion screening was complete, each pair discussed conflicting studies. Disagreements between researchers were resolved by the decision of a third researcher (HG or AR). The 2 sets of included studies were merged and randomly divided into 4 individual sets. Each of the researchers received 1 data set from which to extract the data (described in the Data Extraction section). Most of the data could be extracted from the abstract alone. Therefore, an article’s full text was only reviewed in the event that the information in the abstract was insufficient. If the researcher thought the article was not suitable for inclusion in the study after a more in-depth review, it was excluded.

### Data Extraction

Data extraction was done using a standardized data extraction form (see example in Multimedia Appendix 4) that was developed by AR and HG using Microsoft Office Excel version 16.52. The form was pilot tested by the group of 4 researchers (AR, HG, JM, and PS) on 10 articles and modified afterwards. The data items that were extracted from the articles are described in Table 1. The variable *functionality* was based upon the NICE ESF for DHTs, and the variable *primary user* was based upon the WHO’s digital health classification scheme.

**Table 1:** Data items extracted from the articles.

Generic variables		
		Journal
		Country^a^
		Publication date
		Medical specialty^b^
**Specific variables**		
	Primary user^c^ [3]	Clients
		Health care providers
		Health system or resource managers
		Data services
	Functionality^d^ [4]	System services
		Inform
		Simple monitoring
		Communicate
		Preventative behavior change
		Self-manage
		Treat
		Active monitoring
		Calculate
		Diagnose
		Multiple functionalities
		Other
		Unclear
	Evaluation study phases^e^ [8]	Conceptual and planning
		Design, development, and usability
		Pilot (feasibility)
		Effectiveness (impact)
		Multiple phases
	Evaluation approach	Free-text field

^a^Country in which the study was conducted, or if this was not clear, the country of the first author.

^b^Medical specialty the digital health technology applies to, with multiple options possible; a standardized list was used.

^c^Categories from the World Health Organization classification scheme of digital health interventions were extracted; multiple categories may be extracted.

^d^Categories from the National Institute for Health and Care Excellence’s evidence standards framework were extracted.

^e^Categories from the eHealth evaluation cycles were extracted.

The description of the *evaluation approach* was literally extracted by copying and pasting from the article. If available, multiple evaluation approaches were extracted.

The 4 researchers (AR, HG, JM, and PS) independently extracted the data into the standardized data extraction form. After the data extraction was completed, AR and HG each randomly checked 15 cases from each investigator to evaluate for inconsistencies among the extracted data. When a data item showed an interrater disagreement of more than 10%, a second investigator extracted the data for the specific data item, and a third investigator made the final decision on the inequalities. Considering the extensive workload, this extra step of cross-checking data was performed unblinded. The researchers’ group discussion was planned ahead of the second researcher’s data extraction to increase the level of consensus. Finally, the completed data extraction forms were merged again, checked by AR, and altered for consistency. The description of each of the extracted evaluation approaches was checked and substituted by a better general description to allow for statistical analysis; for example, “randomized controlled trial” was changed to “RCT.”

### Data Synthesis and Analysis

RStudio vs 2022.07.1 software was used to summarize the descriptive data and to perform statistical analyses. The proportions of the categories for each data item are described using percentages. The variable *evaluation approach* was extracted as a free text field in the data extraction form. It was expected that the data extraction for the variable *evaluation approach* would yield a very wide array of different evaluation approaches. Therefore, to design the clearest visual presentation of the data, only the top 8 (cross-tabulation bar chart) and top 10 (bar chart) most frequently used *evaluation approaches* were used for further analysis. Concerning the variable *medical specialty*, the top 5 (cross-tabulation bar chart) and top 10 (bar chart) were used for further analysis. For the variable *country,* only the top 10 (bar chart) were used for analysis.

To examine relationships between the nominal variables, the following cross-tabulations were executed: *primary user* versus *functionality*, *primary user* versus *evaluation study phase*, *primary user* versus top 8 *evaluation approach*, *functionality* versus *evaluation study phase*, *functionality* versus top 8 *evaluation approach*, *evaluation study phase* versus top 5 *medical specialties*, *evaluation study phase* versus top 8 *evaluation approach*, and top 5 *medical specialty* versus top 8 *evaluation approach*.

## Results

**Figure 2:**
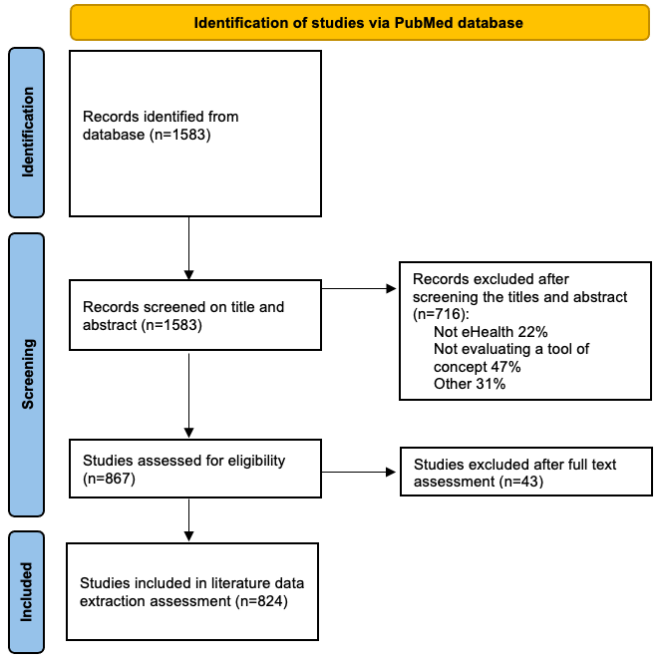
PRISMA (Preferred Reporting Items for Systematic Reviews and Meta-Analyses) flowchart.

After the PubMed database search, 1583 studies were considered relevant according to the inclusion criteria. During the screening of the title and abstract, 716 records were excluded. A further 43 articles were excluded after reading the articles more in depth during the data extraction assessment phase. Finally, 824 studies were included. The PRISMA flowchart summarizes the article selection process (Figure 2).

The independent evaluation of the extracted variables from 15 randomly selected records from each of the 4 researchers by AR and HG revealed unacceptable inconsistencies in the extracted data for the variables *functionality* (27/90, 30%) and *evaluation study phase*s (18/90, 20%). Following a discussion among the 4 researchers, it was decided that the variables *functionality* and *evaluation study phase*s would require a full second review. After the full second review, the variable *functionality* had a mismatch of 29% (250/867), and the variable *evaluation study phases* had a mismatch of 21% (113/867). A third reviewer made the final decision on the discrepancies between the 2 reviewers. All of the researchers participated in the second review, and all made final decisions as a third reviewer.

The top 10 of the general variables *country* and *medical specialty* are shown in Table 2. The complete frequency list of the variables *country*, *medical specialty,* and publishing *journals* from ≥5 studies can be found in Multimedia Appendix 5.

**Table 2:** Top 10 countries and medical specialties in the 824 publications.

Top 10		Results, n (%)
**Country^a^**		
	United States	307 (36.9)
	United Kingdom	63 (7.6)
	Australia	55 (6.6)
	Canada	49 (5.9)
	Netherlands	32 (3.8)
	China	31 (3.7)
	Germany	29 (3.5)
	Spain	20 (2.4)
	Italy	15 (1.8)
	Brazil	15 (1.8)
**Medical specialty^b^**		
	Psychiatry and mental care	89 (10.6)
	Cardiology	75 (8.9)
	Neurology	59 (7)
	Primary care	50 (6)
	Public health	48 (5.7)
	Obstetrics, gynecology, and midwifery	44 (5.2)
	Pediatrics	43 (5.1)
	Endocrinology	38 (4.5)
	Applies to all specialties	36 (4.3)
	Internal medicine	32 (3.8)

^a^Total countries=73.

^b^Total medical specialities=44.

Table 3 shows the descriptive statics of the specific variables. The majority of DHT-related research was published in the field of psychiatry and mental health (89/840, 10.6%) and cardiology (75/840, 8.9%). Concerning DHT *primary user* categories, clients (491/947, 51.8%) were slightly more frequently targeted than health care providers (425/947, 44.9%). Communication (160/824, 19.4%) and treatment (150/824, 18.2%) were the most frequent categories in the *functionality* variable.

**Table 3:** Descriptive statics of specific variables of the studies about digital health technologies (DHTs).

Variables			Frequency, n (%)
**Primary user (n=947 users)**			
	Clients	491 (51.8)	
	Health care providers	425 (44.9)	
	Health system or resource managers	26 (2.7)	
	Data services	5 (0.5)	
**Functionality (n=824 studies)**			
	Communicate	160 (19.4)	
	Treat	150 (18.2)	
	Multiple	103 (12.5)	
	Diagnose	87 (10.6)	
	Self-manage	78 (9.5)	
	Active monitoring	67 (8.1)	
	Preventative behavior change	61 (7.4)	
	Inform	49 (5.9)	
	Simple monitoring	29 (3.5)	
	System services	26 (3.2)	
	Calculate	6 (0.7)	
	Unclear	5 (0.6)	
	Other	3 (0.4)	
**Evaluation study phases (n=824 studies)**			
	Effectiveness (impact)	304 (36.9)	
	Pilot (feasibility)	232 (28.2)	
	Design, development, and usability	99 (12)	
	Conceptual and planning	96 (11.7)	
	Uptake (implementation)	70 (8.5)	
	Multiple phases	23 (2.8)	
**Top 10 evaluation approaches^a^ (n=899)**			
	RCT^b^	170 (18.9)	
	Survey research	91 (10.1)	
	Cohort study (prospective)	73 (8.1)	
	Interview study	58 (6.5)	
	Cohort study (retrospective)	54 (6)	
	Mixed methods study design	42 (4.7)	
	Systematic review	39 (4.3)	
	Cross-sectional study	36 (4)	
	Feasibility study	31 (3.4)	
	Pilot study	25 (2.8)	

^a^108 distinct evaluation approaches were listed.

^b^RCT: randomized controlled trial.

The variable *evaluation study phase* showed that nearly 37% (304/824, 36.9%) of the studies were carried out to study the effectiveness of a certain DHT tool. Almost one-third (232/824, 28.2%) of the studies were in the pilot study phase. RCT was the most frequently used (170/899, 18.9%) *evaluation approach.* In total, 108 distinct *evaluation approaches* were encountered (Multimedia Appendix 6). Although the top 10 consisted of well-known epidemiologic methods, we did encounter novel evaluation methods such as the fit between individual, task, and technology (FITT) framework [17], nonadoption, abandonment, scale-up, spread, and sustainability (NASSS) framework [18], CeHRes Roadmap [19], and systems development life cycle (SDLC) methodology [20]. In addition, several variants of well-known epidemiological methods, such as nonrandomized group comparison study [21] and retrospective record review [22], were identified.

**Figure 3:**
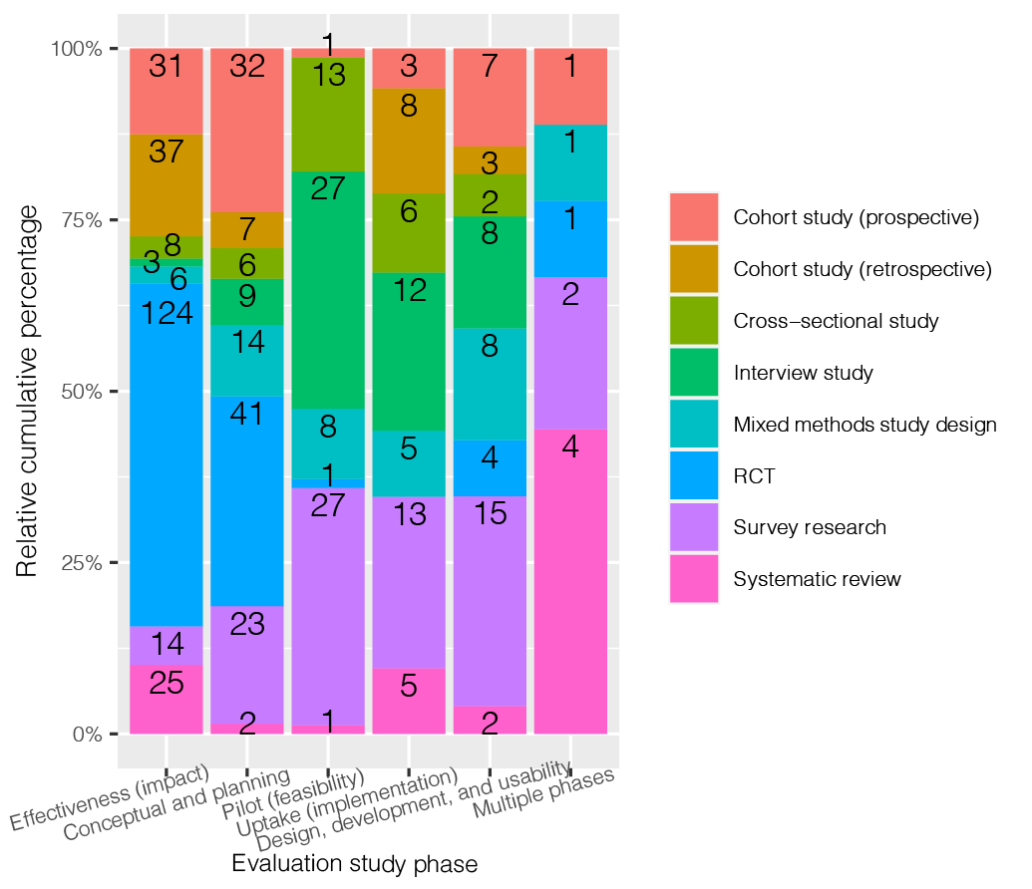
Bar chart evaluation of the study phase versus the top 8 evaluation approaches.

The proportional stacked bar chart in Figure 3 illustrates the cross-tabulation of *evaluation study phase* versus the top 8 *evaluation approaches*. In the effectiveness study phase, RCTs were used in one-half of the studies (124/248, 50%). Prospective and retrospective cohort studies were used in more than one-quarter of the effectiveness studies (68/248, 27.4%). Survey research (27/78, 35%) and interview studies (27/78, 35%) were more commonly used for the conceptual and planning study phase. In the uptake (implementation) study phase, the evaluation approaches used were generally equally divided.

**Figure 4:**
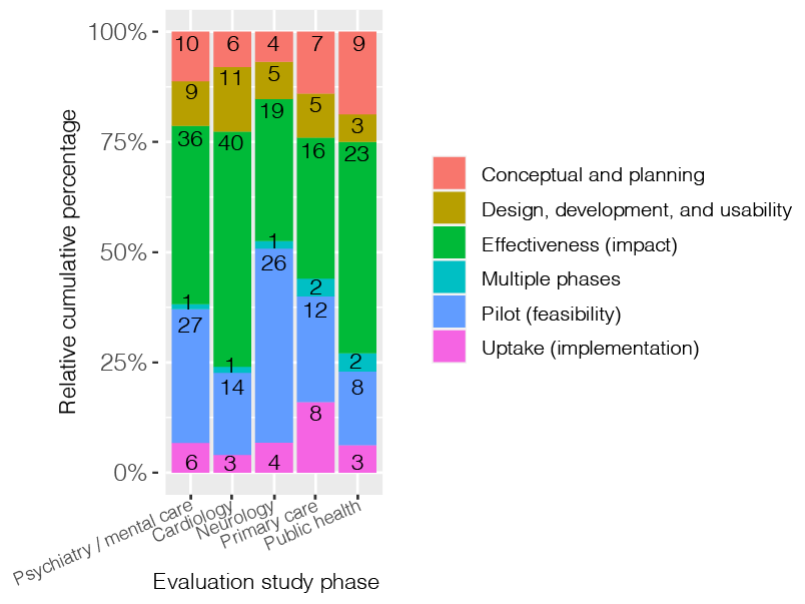
Bar chart evaluation of study phase versus medical specialty.

The proportional stacked bar chart of the cross-tabulation of *evaluation study phase* versus the *top 5 medical specialties* illustrates the differences in emphasis of the selected evaluation study phases between the *medical specialties* (Figure 4). Cardiology had the most studies performed in the effectiveness (impact) study phase (40/75, 54%) and fewer in the pilot study phase (14/75, 19%). Within the medical specialty of neurology, the opposite was found; there was more focus on the pilot study phase (26/59, 44%) and less on the effectiveness study phase (19/59, 32%). Primary care had the most evenly divided chart, with the study phase uptake (implementation; 8/50, 16%) appearing to be of greater importance when compared with other specialties.

**Figure 5:**
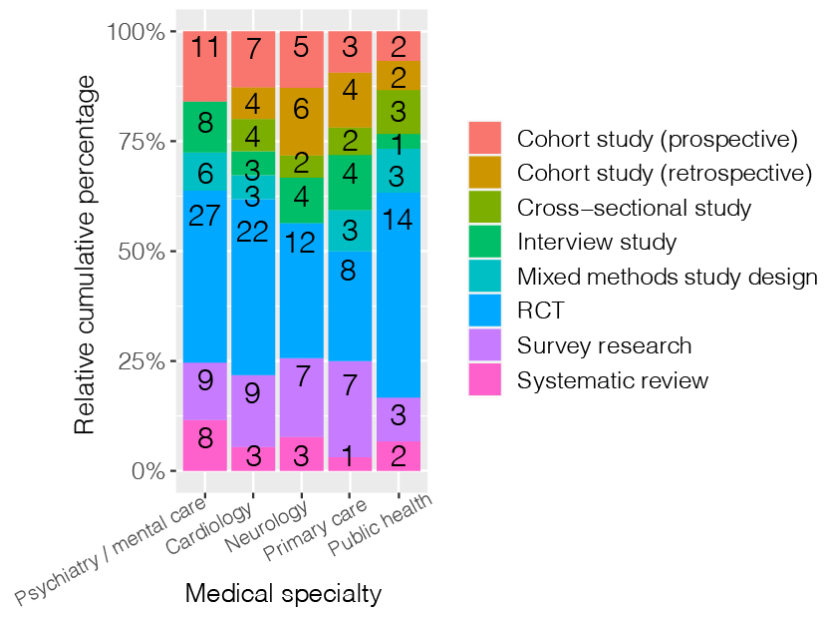
Bar chart evaluation of medical specialty versus the top 8 evaluation approaches.

Further, when looking at the proportional stacked bar chart of the cross-tabulation of *medical specialty* versus the *top 8 evaluation approaches,* the chart shows differences concerning medical specialties and the depicted methodology (Figure 5). The RCT represents the biggest share for all medical specialties, with public health as the front-runner (14/30, 47%). Again, primary care had the most equally divided chart when compared with other specialties.

**Figure 6:**
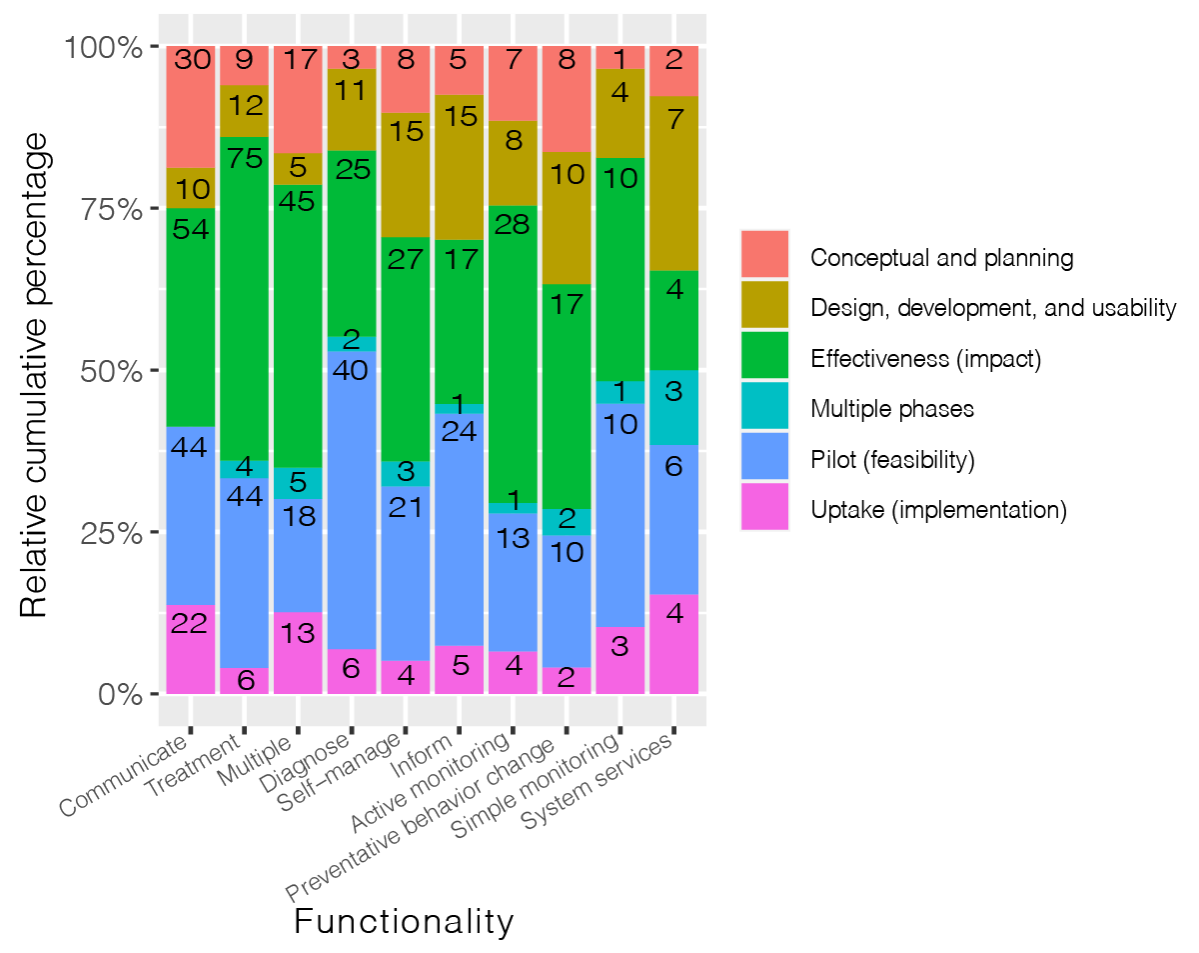
Bar chart evaluation of functionality versus evaluation study phases; the “unclear,” calculate,” and “other” functionality categories were omitted due to limited results.

The cross tabulation of *functionality* versus *evaluation study phase* focuses on the breakdown of the study phase in relation to the assumed DHT function (Figure 6). The effectiveness (impact) study phase was most frequently found in the treatment *functionality* category (75/150, 50%). The pilot (feasibility) study phase was most dominant in the diagnose *functionality* category (40/86, 46%). In almost all categories of the *functionality* variable*,* significantly less attention was paid to the conceptual and planning; design, development, and usability; and uptake (implementation) study phases.

The cross tabulations of *primary user* versus *functionality*, *primary user* versus *evaluation study phase*, *primary user* versus the *top 8 evaluation approaches*, and *functionality* versus the *top 8 evaluation approaches* resulted in an almost equal distribution of the categories. The frequency tables of all the performed cross tabulation analyses, as described in the Methods section, can be found in Multimedia Appendix 7.

## Discussion

**Figure 7:**
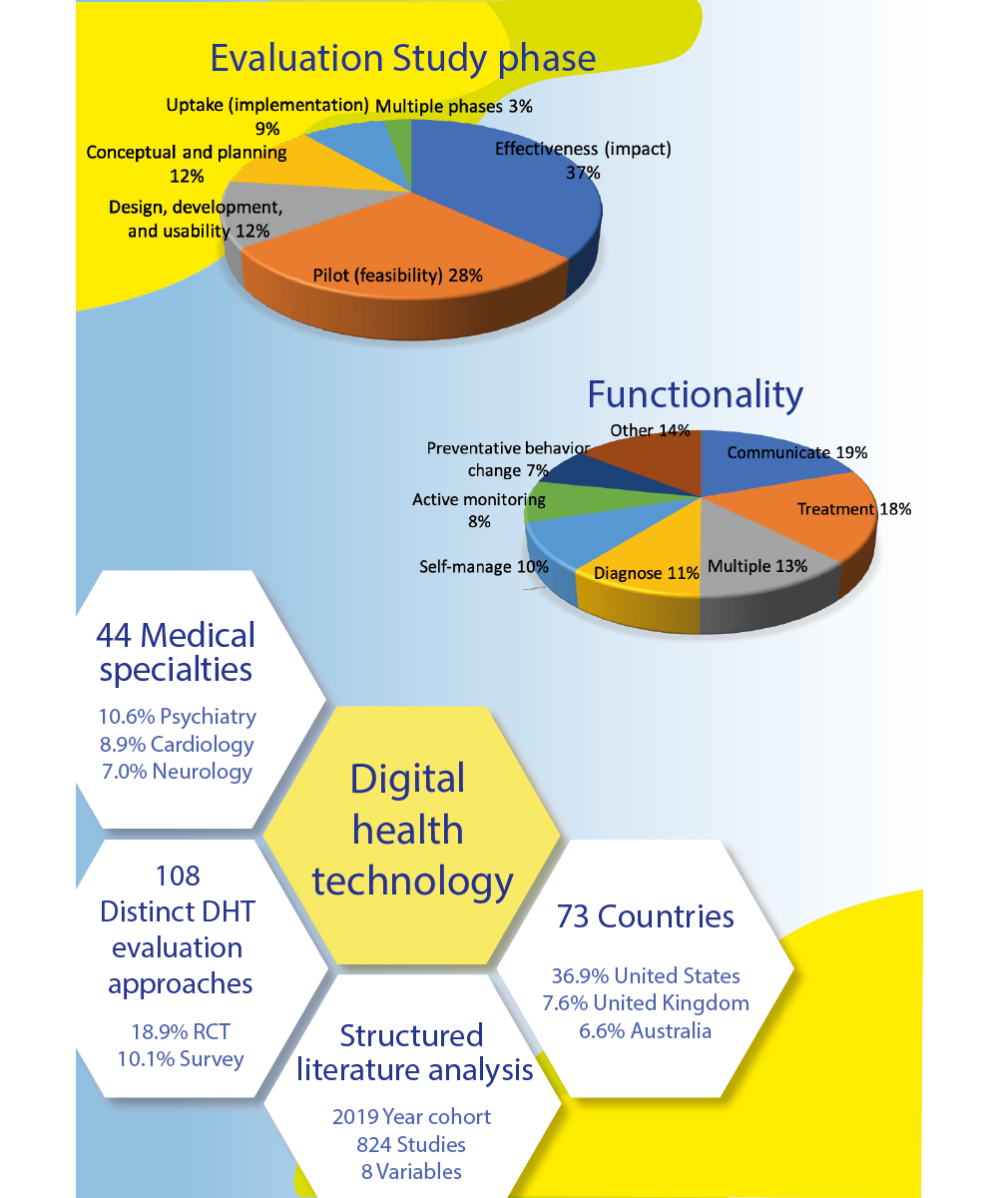
Visual summary of the main findings. RCT: randomized controlled trial.

The aim of this study was to develop a better understanding of the daily practice of the eHealth evaluation cycle. To our knowledge, we composed the first comprehensive overview of the actual practice of the consecutive DHT evaluation study phases. We summarized our main findings in the infographic in Figure 7.

### Evaluation Study Phase and Evaluation Approach

Our study highlighted disparities in the attention given to the consecutive evaluation study phases. A predominant focus on the effectiveness and pilot study phases was found. The uptake phase, crucial for successful implementation and scale-up, received the least emphasis. In addition, less focus has been placed on the planning and development study phases. This fundamental mismatch between the context and the technology is the main reason recognized by the WHO that up to 75% of new medical devices fail [23]. A recently published original study from Royle et al [24] also aimed to create awareness about a lack of evaluation of the methodological steps necessary for developing and testing new clinical care pathways. They proposed “the technology clinical trial,” describing (1) cocreation of care pathways and addressing information governance, data protection, regulatory, and ethical questions by design; (2) delivery of the technology clinical trial; and (3) supporting future research and uptake in practice. This 3-step methodology is comparable to the *eHealth evaluation cycle*; however, the relation of the study phase with DHT evaluation approaches is not described.

The RCT emerged as the most frequently used evaluation approach, posing challenges in the context of rapid and iterative DHT development [25]. We did, however, encounter studies using RCT “subdesigns” such as sequential multiple assignment randomized trial (SMART) and microrandomized trials (Multimedia Appendix 5). These designs allow for limited interventional modification within preset boundaries and therewith might bridge the methodological gap. Recently, Hrynyschyn et al [26] conducted a scoping review aimed at providing an overview of existing evaluation methods of DHTs beyond the RCT. They described in detail microrandomization trials, (fractional) factorial RCTs, SMART, and stepped-wedge cluster randomized trials as promising alternatives.

In the conceptual and planning study phase, survey research and interview studies were each used in one-third of the cases. Although these findings seem obvious, as formative research approaches apply well to the earlier study phases of a development cycle, the importance of thorough “background” research in relation to successful implementation cannot be emphasized enough.

### Functionality (NICE)

In this study, the *functionality* categories of communication and treatment from the NICE’s ESF were the most frequently studied [4,5]. In the *functionality* category of treatment, one-half of the studies focused on the effectiveness (impact) study phase. Concerning the *functionality* category of diagnose, the majority of the studies were found in the pilot (feasibility) study phase. DHTs in the treatment and diagnose categories are both considered “tier 3b” according to the ESF. This implies that designed DHTs must demonstrate effectiveness through a high-quality intervention study (experimental or quasiexperimental design) showing improvements in relevant outcomes. Therefore, it seems to make sense that the majority of the studies focused on the pilot (feasibility) and effectiveness (impact) study phases. Unfortunately, there seems to be too little attention paid to the predevelopment and postdeployment phases, which are also not included in the NICE’s ESF.

DHTs are often developed by commercial entities, which then need validation through research to allow for regulatory clearance by governing bodies (eg, the Food and Drug Administration [FDA]) and subsequent adoption and commercialization. Hence, they may only pursue research following the development of a DHT, allowing for speed and rapid prototyping unhindered by slow academic rigor. Often, the development phase involves some form of user testing and prototyping with feedback; however, the value in publishing this is less pronounced and perhaps even avoided to protect intellectual property and avoid competition before commercialization. Despite the time limitations and stakeholder involvement required, perhaps greater attention should be placed on evaluating DHTs focused on treatment in the earlier design and conceptual phases to improve success and uptake at later phases (in terms of costs, usefulness, and adoption, for example). The CeHRes roadmap is one proposed approach to the development of eHealth interventions. It incorporates both a human-centered design and a business model focus, to create potentially value-adding and sustainable eHealth technologies [27].

### Primary User (WHO)

The target end users of studies, as described by the WHO, were primarily focused on clients and health care providers. Health systems or resource managers and data services accounted for less than 4% of studies. This might be due to the limitation of the search to the clinical database PubMed. For example, the IEEE database perhaps would have yielded more relevant results. However, it also might suggest that the majority of technological innovation and implementation is focused on providers and clients (ie, at the point of care rather than at the system and infrastructure levels). This is understandable, considering that health organizations have a level of complexity and fragmented structures that constrain their ability to adopt organization-wide digitization [28,29]. As such, a recent study described how to improve and manage sustainable hybrid (eHealth and face-to-face) health care on an organizational level through the use of the Hybrid Health Care Quality Assessment [30]. The other consideration regarding target end users is the rate of adoption, which is a big contributor to successful implementation and uptake of health technology. Providers remain, to a large extent, the gatekeepers of this, as health care professionals’ acceptance is reported as an important requirement for the success of clinical systems [31]. This could explain why the majority of DHTs and their evaluation studies are focused on providers and clients.

### Countries and Medical Specialties

The United States published the largest number of DHT evaluation studies. The number of studies per country aligns with the overall ranking of health publications in general per country being the United States, the United Kingdom, Australia, and Canada. However, China, which usually features in third place, featured slightly lower, in sixth place, and the Netherlands placed significantly higher, moving to fifth from 13th place [32]. These discrepancies may be explained by the fact that our study excluded Chinese databases or non-English papers. The numbers for the Netherlands suggest that there is a greater attention to eHealth evaluations in general. According to the Healthcare Information and Management Systems Society (HIMSS), the Netherlands is one of the front-runners in the digitalization of health care [33].

Psychiatry and mental health was the most frequently encountered medical specialty involved in DHT research, followed by cardiology and neurology. A 2018 US-based, weighted survey on the adoption of DHT by physicians in different specialties showed a varied spectrum of adoption rates. Consistent with the data we collected in this scoping review, 27.8% of psychiatrists and 24.1% of cardiologists used DHT for patient interactions [34]. DHTs for mental health have seen increased proliferation, considering inherent benefits such as anonymity, accessibility, and acceptability, possibly explaining why this is the leading specialty in DHT research studies [35].

When relating the medical specialties to the evaluation study phases, the medical specialties of cardiology and neurology showed interestingly opposing results. Of the included cardiology studies, one-half were in the effectiveness phase, and less than one-fifth were in the pilot study phase. As for neurology studies, one-third of the studies were in the effectiveness phase, and almost one-half were in the pilot study phase. As such, perhaps there is less focus on the pilot phase within cardiology, and fewer neurology studies progress to the effectiveness study phase. This could also be an indication of the stage of technological development and application in each specialty and the year of publication we evaluated (2019). For example, technology has been used in cardiology since the first implantable pacemaker in 1958, with studies on topics such as remote monitoring and virtual reality surfacing between 2000 and 2010, and technology has sustained considerable scholarly attention ever since [36,37]. Whereas in neurology, for example, there are very few papers on fall detection using the Internet of Things before 2010, as this is a relatively newer field of study [38].

### Limitations

Although our scoping review was thorough, there are some limitations to mention. We only searched in the PubMed database and included only English, peer-reviewed literature, which excludes studies based in foreign contexts (eg, Chinese as well as grey literature), which are used by a large number of commercial institutions involved in the development of DHTs. However, this would have had questionable value regarding methodology and scientific rigor. Only papers published in 2019 were considered in the data extraction step. The decision to include studies from 2019 was made in order to have the most up-to-date data available, without considering the impact of the COVID-19 pandemic on DHT research. Because we only extracted data from studies published in 2019, we might have missed novel evaluation approaches. Further, a possible shift in the evaluation approaches used over time could not be evaluated. It would be interesting to conduct comparisons between time points in a future study (eg, 2015 vs 2019 vs 2023 cohort).

Due to unacceptable discrepancies between the researchers when including data during the data extraction phase for the variables *functionality* and *evaluation study phase*, a second unblinded cross-checking was performed to facilitate concordance, which may have introduced a level of bias. Further, during data extraction, the reviewers standardized the terminology for evaluation approaches, which is open to a level of bias in interpretation. However, this should be minimal considering research uses common terminology in general.

Unfortunately, we were not able to include a variable describing the DHT (eg, “patient portal” or “mHealth” [mobile health]). In the majority of the included studies, the description of the DHT varied too much. Therefore, more in-depth (sub)analyses concerning the type of DHT were not possible. In our opinion, a well-developed, easy-to-use DHT taxonomy would be of great help to classify and evaluate future DHT research. Last, the NICE ESF for DHT was updated in August 2022. However, the majority the subgroups in our study are still used in the NICE framework.

### Future

It would be valuable to track which technologies are evaluated in a single study phase or in multiple study phases. However, to do this, each technology would require unique identification that would allow traceability across studies. This would allow visibility on which technologies that begin from a conceptual evaluation phase are eventually implemented and evaluated in subsequent phases for effectiveness and uptake. It would also provide clarity on how many technologies remain in the research stage and fail to see practical clinical implementation.

The results of this study may be used to pinpoint areas of DHT research that require more focus and support the completion of multiple steps of the *eHealth evaluation cycle*. In such, future research could also look at a temporal view of research in relation to medical specialty and evaluation phase, as this could provide further insights as to the stage of digital transformation and where certain fields are lagging behind.

The Excel data file (see the Data Availability section) with the extracted data from the 824 included studies is published online [9,39]. We encourage interested researchers to use the filter options to look at subsets of data for the field of interest. For example, when one is interested in cardiology studies in the pilot phase, one can select “pilot” study phase, and cardiology studies within the pilot study phase from 2019 will be shown. Finally, all encountered evaluation approaches are published in an online freely accessible document (Multimedia Appendix 6). The document will be updated on a regular basis, and we encourage readers of this paper to email additions to the document if any evaluation approach is missing.

### Conclusion

Improving the success of the development and implementation of DHTs is crucial to enhancing the transition to an efficient and future-proofed health care system. We developed a better understanding of the practice of the sequential evaluation study phases of the eHealth evaluation cycle and the use of the relative DHT evaluation approaches. In the *eHealth evaluation cycle*, most attention is paid to the pilot (feasibility) and effectiveness (impact) study phases. Whether the evaluation of the earlier study phases and the uptake (implementation) study phase indeed improve successful outcomes is yet to be evaluated. Surveys, interviews, and mixed methods dominated the earlier study phases of DHT research. Although the majority of the evaluation approaches still used an RCT design, the iterative nature of technology may be better suited to more novel assessment approaches. The most often explored DHTs were those focusing on treatment and communication. Interestingly, the specialties of psychiatry and mental health, cardiology, and neurology were more interested in DHT evaluation than others. This offers potential opportunities to focus on unaddressed specialties in the search for DHTs, which can provide novel ways to transform and improve our health care system. Finally, future research might benefit from tracking and sharing which technologies successfully proceed through all stages of the *eHealth evaluation cycle*.

## Appendix

Multimedia Appendix 1 World Health Organization (WHO) classification scheme.

Multimedia Appendix 2 PRISMA-ScR (Preferred Reporting Items for Systematic Reviews and Meta-Analyses Extension for Scoping Reviews) checklist.

Multimedia Appendix 3 PubMed search.

Multimedia Appendix 4 Data extraction sheet.

Multimedia Appendix 5 Results variables country, medical specialty and journals.

Multimedia Appendix 6 Complete list of evaluation approaches.

Multimedia Appendix 7 All performed cross tabulations.

## Abbreviations

DHT: digital health technology
ESF: Evidence Standards Framework
FDA: Food and Drug Administration
FITT: fit between individual, task, and technology
HIMSS: Healthcare Information and Management Systems Society
mHealth: mobile health
NASSS: nonadoption, abandonment, scale-up, spread, and sustainability
NICE: National Institute for Health and Care Excellence
PRISMA: Preferred Reporting Items for Systematic Reviews and Meta-Analyses
RCT: randomized controlled trial
SDLC: systems development life cycle
SMART: sequential multiple assignment randomized trial
WHO: World Health Organization

## Data Availability

The data sets generated during and/or analyzed during this study are available in the online “Evaluation Approaches of Digital Health Technologies” [40].

## References

[1] (2022). Ageing and health. World Health Organization.

[2] Lau F, Kuziemsky C, Lau F, Kuziemsky C (2017). What is eHealth?. Handbook of eHealth Evaluation: An Evidence-based Approach.

[3] (2018). Classification of digital health interventions v1.0. World Health Organization.

[4] (2019). Evidence Standards Framework for Digital Health Technologies Contents. National Institute for Health and Care Excellence.

[5] Unsworth H, Dillon B, Collinson L, Powell H, Salmon M, Oladapo T, Ayiku L, Shield G, Holden J, Patel N, Campbell M, Greaves F, Joshi I, Powell J, Tonnel A (2021). The NICE Evidence Standards Framework for digital health and care technologies - Developing and maintaining an innovative evidence framework with global impact. Digit Health.

[6] Campbell M, Fitzpatrick R, Haines A, Kinmonth AL, Sandercock P, Spiegelhalter D, Tyrer P (2000). Framework for design and evaluation of complex interventions to improve health. BMJ.

[7] Craig P, Dieppe P, Macintyre S, Michie S, Nazareth I, Petticrew M, Medical Research Council Guidance (2008). Developing and evaluating complex interventions: the new Medical Research Council guidance. BMJ.

[8] Bonten TN, Rauwerdink A, Wyatt JC, Kasteleyn MJ, Witkamp L, Riper H, van Gemert-Pijnen LJ, Cresswell K, Sheikh A, Schijven MP, Chavannes NH, EHealth Evaluation Research Group (2020). Online guide for electronic health evaluation approaches: systematic scoping review and concept mapping study. J Med Internet Res.

[9] eHealth methodology guide. Citrienprogramma e-health.

[10] Trochim W, Kane M (2005). Concept mapping: an introduction to structured conceptualization in health care. Int J Qual Health Care.

[11] Safavi K, Cohen A, Ting D, Chaguturu S, Rowe J (2020). Health systems as venture capital investors in digital health: 2011-2019. NPJ Digit Med.

[12] Maramba I, Chatterjee A, Newman C (2019). Methods of usability testing in the development of eHealth applications: A scoping review. Int J Med Inform.

[13] Ross J, Stevenson F, Lau R, Murray E (2016). Factors that influence the implementation of e-health: a systematic review of systematic reviews (an update). Implement Sci.

[14] Tomlinson M, Rotheram-Borus MJ, Swartz L, Tsai AC (2013). Scaling up mHealth: where is the evidence?. PLoS Med.

[15] Aminoff H, Meijer S (2021). Context and complexity in telemedicine evaluation: work domain analysis in a surgical setting. JMIR Perioper Med.

[16] Rayyan.

[17] Cho H, Flynn G, Saylor M, Gradilla M, Schnall R (2019). Use of the FITT framework to understand patients' experiences using a real-time medication monitoring pill bottle linked to a mobile-based HIV self-management app: A qualitative study. Int J Med Inform.

[18] Dijkstra A, Heida A, van Rheenen PF (2019). Exploring the challenges of implementing a web-based telemonitoring strategy for teenagers with inflammatory bowel disease: empirical case study. J Med Internet Res.

[19] van Gemert-Pijnen JEWC, Nijland N, van Limburg M, Ossebaard HC, Kelders SM, Eysenbach G, Seydel ER (2011). A holistic framework to improve the uptake and impact of eHealth technologies. J Med Internet Res.

[20] Kho SES, Lim SG, Hoi WH, Ng PL, Tan L, Kowitlawakul Y (2019). The development of a diabetes application for patients with poorly controlled type 2 diabetes mellitus. Comput Inform Nurs.

[21] Dial HR, Hinshelwood HA, Grasso SM, Hubbard HI, Gorno-Tempini M, Henry ML (2019). Investigating the utility of teletherapy in individuals with primary progressive aphasia. Clin Interv Aging.

[22] Hoth AB, Shafer C, Dillon DB, Mayer R, Walton G, Ohl ME (2019). Iowa TelePrEP: a public-health-partnered telehealth model for human immunodeficiency virus preexposure prophylaxis delivery in a rural state. Sex Transm Dis.

[23] WHO Medical devices: Managing the Mismatch: An outcome of the Priority Medical Devices project 2010. World Health Organization.

[24] Royle JK, Hughes A, Stephenson L, Landers D (2021). Technology clinical trials: Turning innovation into patient benefit. Digit Health.

[25] Guo C, Ashrafian H, Ghafur S, Fontana G, Gardner C, Prime M (2020). Challenges for the evaluation of digital health solutions-A call for innovative evidence generation approaches. NPJ Digit Med.

[26] Hrynyschyn R, Prediger C, Stock C, Helmer SM (2022). Evaluation methods applied to digital health interventions: what is being used beyond randomised controlled trials?-a scoping review. Int J Environ Res Public Health.

[27] Van Velsen L, Wentzel J, Van Gemert-Pijnen JE (2013). Designing eHealth that matters via a multidisciplinary requirements development approach. JMIR Res Protoc.

[28] England I, Stewart D, Walker S (2000). Information technology adoption in health care: when organisations and technology collide. Aust Health Rev.

[29] Golinelli D, Boetto E, Carullo G, Nuzzolese AG, Landini MP, Fantini MP (2020). Adoption of digital technologies in health care during the COVID-19 pandemic: systematic review of early scientific literature. J Med Internet Res.

[30] Tossaint-Schoenmakers R, Kasteleyn MJ, Rauwerdink A, Chavannes N, Willems S, Talboom-Kamp EPWA (2022). Development of a quality management model and self-assessment questionnaire for hybrid health care: concept mapping study. JMIR Form Res.

[31] Esmaeilzadeh P, Sambasivan M, Kumar N, Nezakhati H (2011). Adoption of technology applications in healthcare: the influence of attitude toward knowledge sharing on technology acceptance in a hospital. U- and E-Service, Science and Technology.

[32] Scimago.

[33] HIMSS Annual European Digital Health Survey. Healthcare Information and Management Systems Society, Inc.

[34] Kane CK, Gillis K (2018). The use Of telemedicine by physicians: still the exception rather than the rule. Health Aff (Millwood).

[35] Basnet S (2014). The feasibility of eHealth in mental health care. J Addict Res Ther.

[36] van Hemel NM, van der Wall EE (2008). 8 October 1958, D Day for the implantable pacemaker. Neth Heart J.

[37] Zwack C, Haghani M, Hollings M, Zhang L, Gauci S, Gallagher R, Redfern J (2023). The evolution of digital health technologies in cardiovascular disease research. NPJ Digit Med.

[38] Newaz NT, Hanada E (2023). The methods of fall detection: a literature review. Sensors (Basel).

[39] Projects. NeLL.

[40] Database Evaluation Approaches of Digital Health Technologies.

