# Supplementary figures and images for: Approaches to Evaluating Digital Health Technologies: Scoping Review
Source: J Med Internet Res. 2024 Aug 28;26:e50251. doi: 10.2196/50251 (PMC11391152; doi:10.2196/50251)

Multimedia Appendix 1. WHO classification scheme of digital health interventions


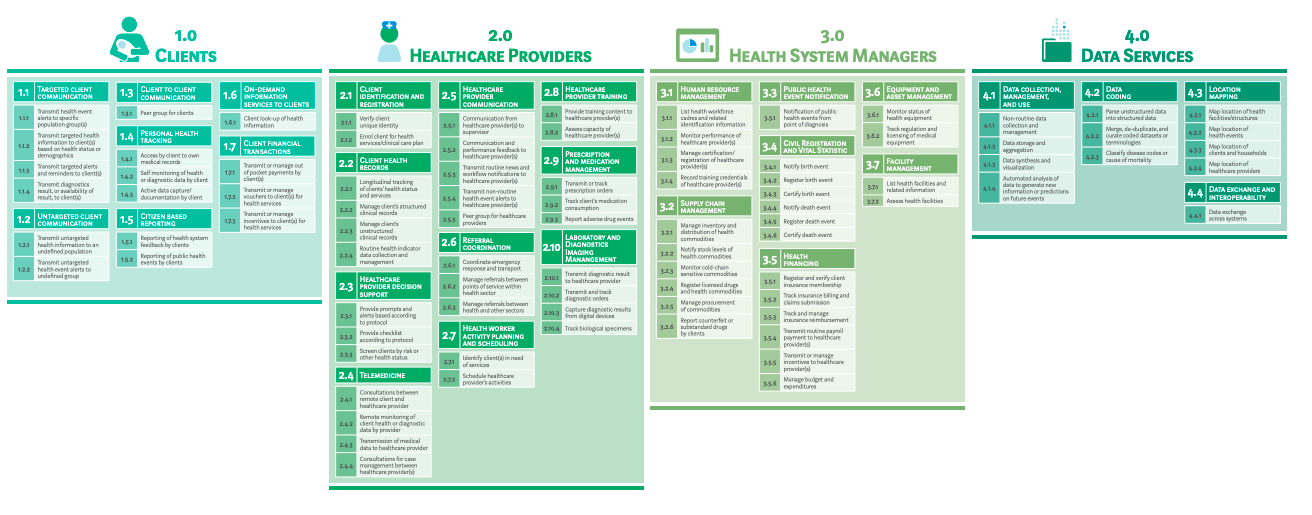

Supplement: Multimedia Appendix 1 [file jmir_v26i1e50251_app1.docx]
