# Supplementary material for: Approaches to Evaluating Digital Health Technologies: Scoping Review
Source: J Med Internet Res. 2024 Aug 28;26:e50251. doi: 10.2196/50251 (PMC11391152; doi:10.2196/50251)
Supplement: Multimedia Appendix 3 [file jmir_v26i1e50251_app3.docx]

Multimedia Appendix 1

Search 26/8/2020 | Hits: 1583

Database: PubMed

((epidemiological Stud*[tiab] OR epidemiologic Stud*[tiab] OR case-control Stud*[tiab] OR case control Stud*[tiab] OR Case series[tiab] OR cohort Stud*[tiab] OR cohort Analy*[tiab] OR prospective Stud*[tiab] OR retrospective stud*[tiab] OR longitudinal Stud*[tiab] OR controlled before-after Stud*[tiab] OR controlled before after Stud*[tiab] OR (controlled before and after Stud*[tiab]) OR controlled before-and-after Stud*[tiab] OR CBA Stud*[tiab] OR before-and-after Stud*[tiab] OR (before and after Stud*[tiab]) OR controlled clinical Trial*[tiab] OR cross sectional Stud*[tiab] OR cross-sectional Stud*[tiab] OR cross sectional Analy*[tiab] OR cross-sectional Analy*[tiab] OR Interrupted Time Series[tiab] OR ITS Stud*[tiab] OR feasibility Stud*[tiab] OR pilot Stud*[tiab] OR Trial design[tiab] OR Study design[tiab] OR clinical Trial*[tiab] OR adaptive clinical Trial*[tiab] OR non randomized controlled Trial*[tiab] OR non-randomized controlled Trial*[tiab] OR nonrandomized clinical Trial*[tiab] OR nonrandomized controlled Trial*[tiab] OR non randomized clinical Trial*[tiab] OR non-randomized clinical Trial*[tiab] OR Cluster randomized controlled trial[tiab] OR non randomised controlled Trial*[tiab] OR non-randomised controlled Trial*[tiab] OR non randomized clinical Trial*[tiab] OR non randomised controlled Trial*[tiab] OR non randomized clinical Trial*[tiab] OR non randomized clinical Trial*[tiab] OR Cluster randomised controlled trial[tiab] OR quasi-experimental Stud*[tiab] OR quasi experimental Stud*[tiab] OR experimental stud*[tiab] OR superiority Trial*[tiab] OR non inferiority Trial*[tiab] OR non inferiority Trial*[tiab] OR noninferiority Trial*[tiab] OR Non-inferiority trial[tiab] OR pragmatic Trial*[tiab] OR multicentre Stud*[tiab] OR multicenter Trial*[tiab] OR multicentre Trial*[tiab] OR multicentre Stud*[tiab] OR focus group stud*[tiab] OR interview stud*[tiab] OR survey stud*[tiab] OR Action research[tiab] OR A/B testing[tiab] OR Adaptive design[tiab] OR big data analy*[tiab] OR CeHRes[tiab] OR CHEATS[tiab] OR cognitive task analy*[tiab] OR Cognitive walkthrough[tiab] OR Concept mapping[tiab] OR CEEBIT[tiab] OR cost-effectiveness analy*[tiab] OR cost effectiveness analy*[tiab] OR Critical incident technique[tiab] OR Economic evaluation[tiab] OR eASI[tiab] OR eHealth Needs Assessment Questionnaire[tiab] OR e-Health Needs Assessment Questionnaire[tiab] OR Evaluative Questionnaire for E-health Tools[tiab] OR Evaluative Questionnaire for Ehealth Tools[tiab] OR Five-stage model for comprehensive research on telehealth[tiab] OR Fractional-factorial design[tiab] OR Fractional factorial design[tiab] OR factorial ANOVA design[tiab] OR (HAS methodological framework[tiab]) OR (HAS framework[tiab]) OR Heuristic evaluation[tiab] OR Life-cycle-based approach[tiab] OR Living lab[tiab] OR log file analy*[tiab] OR log file analy*[tiab] OR Matched cohort study design[tiab] OR Method for technology-delivered Healthcare Measures[tiab] OR Methods comparison study[tiab] OR (mHealth Agile and User-Centered Research and Development Lifecycle[tiab]) OR (mHealth Development and Evaluation Framework[tiab]) OR Micro-randomized trial[tiab] OR Micro-randomised trial[tiab] OR Micro randomised trial[tiab] OR Micro randomized trial[tiab] OR Mixed methods[tiab] OR Model for Assessment of Telemedicine applications[tiab] OR MAST[tiab] OR Model of Fog[tiab] OR Model of Oinas-Kukkonen[tiab] OR Multiphase Optimization Strategy[tiab] OR Multiphase Optimisation Strategy[tiab] OR Normalization process model[tiab] OR Normalisation process model[tiab] OR Parallel cohort design with nested RCT[tiab] OR Participatory design[tiab] OR Patient reported outcome measures[tiab] OR PROMs[tiab] OR Practical clinical trial[tiab] OR Preference clinical trial[tiab] OR Pretest-posttest design[tiab] OR Pretest posttest design[tiab] OR Propensity score[tiab] OR Framework for Evaluating mHealth Services[tiab] OR RE-AIM[tiab] OR Simulation study[tiab] OR Single-case experiment[tiab] OR Single case experiment[tiab] OR N=1[tiab] OR N of 1[tiab] OR N-of-1[tiab] OR Sociotechnical evaluation[tiab] OR Stage Model of Behavioral Therapies Research[tiab] OR Stead's et al. evaluation framework[tiab] OR Stepped wedge trial design[tiab] OR Technology Acceptance Model[tiab] OR Think aloud[tiab] OR Trials of intervention principles[tiab] OR User-centered design[tiab] OR User centered design[tiab] OR User-based evaluation[tiab] OR User based evaluation[tiab] OR Vignette study[tiab] OR Wait list control group design[tiab] OR review[tiab] OR "Health Care Evaluation Mechanisms"[Mesh] OR "Evaluation Studies as Topic"[Mesh] OR "Implementation Science"[Mesh])

AND (Telemedicine [mesh])

AND (2019/01/01:2019/12/31[dp] AND english[Filter]))

NOT (protocol[tiab] OR proposal[tiab] OR opinion paper[tiab] OR opinion article[tiab] OR critical view[tiab])
