# Supplementary material for: Approaches to Evaluating Digital Health Technologies: Scoping Review
Source: J Med Internet Res. 2024 Aug 28;26:e50251. doi: 10.2196/50251 (PMC11391152; doi:10.2196/50251)
Supplement: Multimedia Appendix 5 [file jmir_v26i1e50251_app5.docx]

Multimedia appendix 3

Country

|  | **Country** | **Frequency** | **Percentage** |
| --- | --- | --- | --- |
| 1 | United States | 307 | 36.9% |
| 2 | United Kingdom | 63 | 7.6% |
| 3 | Australia | 55 | 6.6% |
| 4 | Canada | 49 | 5.9% |
| 5 | Netherlands | 32 | 3.8% |
| 6 | China | 31 | 3.7% |
| 7 | Germany | 29 | 3.5% |
| 8 | Spain | 20 | 2.4% |
| 9 | Brazil | 15 | 1.8% |
| 10 | Italy | 15 | 1.8% |
| 11 | India | 14 | 1.7% |
| 12 | Sweden | 12 | 1.4% |
| 13 | Denmark | 11 | 1.3% |
| 14 | Norway | 11 | 1.3% |
| 15 | Kenya | 10 | 1.2% |
| 16 | Austria | 9 | 1.1% |
| 17 | Bangladesh | 8 | 1.0% |
| 18 | France | 8 | 1.0% |
| 19 | Israel | 8 | 1.0% |
| 20 | South Africa | 8 | 1.0% |
| 21 | Iran, Islamic Rep. | 7 | 0.8% |
| 22 | Japan | 7 | 0.8% |
| 23 | Switzerland | 7 | 0.8% |
| 24 | Belgium | 5 | 0.6% |
| 25 | Korea, Rep. | 5 | 0.6% |
| 26 | Taiwan | 5 | 0.6% |
| 27 | Thailand | 5 | 0.6% |
| 28 | Ghana | 4 | 0.5% |
| 29 | Ireland | 4 | 0.5% |
| 30 | Korea, Dem. People's Rep. | 4 | 0.5% |
| 31 | Poland | 4 | 0.5% |
| 32 | Singapore | 4 | 0.5% |
| 33 | Finland | 3 | 0.4% |
| 34 | New Zealand | 3 | 0.4% |
| 35 | Uganda | 3 | 0.4% |
| 36 | Argentina | 2 | 0.2% |
| 37 | Chile | 2 | 0.2% |
| 38 | Colombia | 2 | 0.2% |
| 39 | Ethiopia | 2 | 0.2% |
| 40 | Hong Kong SAR, China | 2 | 0.2% |
| 41 | Malawi | 2 | 0.2% |
| 42 | Mexico | 2 | 0.2% |
| 43 | Portugal | 2 | 0.2% |
| 44 | Senegal | 2 | 0.2% |
| 45 | Turkey | 2 | 0.2% |
| 46 | Albania | 1 | 0.1% |
| 47 | Bosnia and Herzegovina | 1 | 0.1% |
| 48 | Burkina Faso | 1 | 0.1% |
| 49 | Cameroon | 1 | 0.1% |
| 50 | Czech Republic | 1 | 0.1% |
| 51 | Ecuador | 1 | 0.1% |
| 52 | Greenland | 1 | 0.1% |
| 53 | Guinea | 1 | 0.1% |
| 54 | Haiti | 1 | 0.1% |
| 55 | Hungary | 1 | 0.1% |
| 56 | Indonesia | 1 | 0.1% |
| 57 | Latin America & Caribbean | 1 | 0.1% |
| 58 | Lithuania | 1 | 0.1% |
| 59 | Madagascar | 1 | 0.1% |
| 60 | Malaysia | 1 | 0.1% |
| 61 | Morocco | 1 | 0.1% |
| 62 | Nepal | 1 | 0.1% |
| 63 | Niger | 1 | 0.1% |
| 64 | Nigeria | 1 | 0.1% |
| 65 | Pakistan | 1 | 0.1% |
| 66 | Saudi Arabia | 1 | 0.1% |
| 67 | Slovenia | 1 | 0.1% |
| 68 | Tanzania | 1 | 0.1% |
| 69 | Timor-Leste | 1 | 0.1% |
| 70 | Unclear | 1 | 0.1% |
| 71 | Vietnam | 1 | 0.1% |
| 72 | Zambia | 1 | 0.1% |
| 73 | Zimbabwe | 1 | 0.1% |

Medical specialty

|  | **Medical specialty** | **Frequency** | **Percentage** |
| --- | --- | --- | --- |
| 1 | Psychiatry / mental care | 89 | 10.6% |
| 2 | Cardiology | 75 | 8.9% |
| 3 | Neurology | 59 | 7.0% |
| 4 | Primary care | 50 | 6.0% |
| 5 | Public Health | 48 | 5.7% |
| 6 | Obstetrics and gynaecology and midwifery | 44 | 5.2% |
| 7 | Paediatrics | 43 | 5.1% |
| 8 | Endocrinology | 38 | 4.5% |
| 9 | Applies to all specialties | 36 | 4.3% |
| 10 | Internal medicine | 32 | 3.8% |
| 11 | Physical medicine and rehabilitation | 30 | 3.6% |
| 12 | Oncology | 25 | 3.0% |
| 13 | Ophthalmology | 24 | 2.9% |
| 14 | Pulmonology | 24 | 2.9% |
| 15 | General surgery | 22 | 2.6% |
| 16 | Infectious disease | 22 | 2.6% |
| 17 | Dermatology-Venereology | 21 | 2.5% |
| 18 | Geriatrics | 18 | 2.1% |
| 19 | Orthopaedics | 16 | 1.9% |
| 20 | Otorhinolaryngology | 16 | 1.9% |
| 21 | Emergency medicine | 14 | 1.7% |
| 22 | Gastroenterology | 13 | 1.5% |
| 23 | Urology | 10 | 1.2% |
| 24 | General practice | 9 | 1.1% |
| 25 | Nutrition | 8 | 1.0% |
| 26 | Intensive care medicine | 7 | 0.8% |
| 27 | pharmacology | 6 | 0.7% |
| 28 | Allergy and immunology | 5 | 0.6% |
| 29 | Anaesthesiology | 4 | 0.5% |
| 30 | Nephrology | 4 | 0.5% |
| 31 | Oral and maxillofacial surgery | 4 | 0.5% |
| 32 | Plastic, reconstructive and aesthetic surgery | 4 | 0.5% |
| 33 | Multiple | 3 | 0.4% |
| 34 | Neonatology | 3 | 0.4% |
| 35 | Neurosurgery | 3 | 0.4% |
| 36 | Paediatric surgery | 2 | 0.2% |
| 37 | Pathology | 2 | 0.2% |
| 38 | cardiology | 1 | 0.1% |
| 39 | Dental care | 1 | 0.1% |
| 40 | Haematology | 1 | 0.1% |
| 41 | obstetrics and gynaecology and midwifery | 1 | 0.1% |
| 42 | Pharmacy | 1 | 0.1% |
| 43 | Radiation Oncology | 1 | 0.1% |
| 44 | Vascular | 1 | 0.1% |

List of journals from 5 studies and up^*^

|  | **Name of journal** | **Frequency (n)** | **Percentage** |
| --- | --- | --- | --- |
| 1 | Journal of medical Internet research | 47 | 5.7% |
| 2 | JMIR mHealth and uHealth | 35 | 4.2% |
| 3 | Telemedicine journal and e-health : the official journal of the American           Telemedicine Association | 35 | 4.2% |
| 4 | Journal of telemedicine and telecare | 33 | 4.0% |
| 5 | PloS one | 19 | 2.3% |
| 6 | Studies in health technology and informatics | 15 | 1.8% |
| 7 | International journal of environmental research and public health | 11 | 1.3% |
| 8 | BMC medical informatics and decision making | 10 | 1.2% |
| 9 | Translational behavioral medicine | 10 | 1.2% |
| 10 | BMJ open | 9 | 1.1% |
| 11 | BMC health services research | 7 | 0.8% |
| 12 | BMC public health | 7 | 0.8% |
| 13 | International journal of medical informatics | 7 | 0.8% |
| 14 | Journal of medical systems | 7 | 0.8% |
| 15 | Computers; informatics; nursing : CIN | 6 | 0.7% |
| 16 | AIDS and behavior | 5 | 0.6% |
| 17 | Circulation. Cardiovascular quality and outcomes | 5 | 0.6% |
| 18 | Journal of affective disorders | 5 | 0.6% |
| 19 | Journal of substance abuse treatment | 5 | 0.6% |
| 20 | Journal of the American Pharmacists Association : JAPhA | 5 | 0.6% |
| 21 | Schizophrenia research | 5 | 0.6% |
| 22 | The British journal of general practice : the journal of the Royal College of           General Practitioners | 5 | 0.6% |

*Total number of included distinct journals n = 418
