# Supplementary material for: Approaches to Evaluating Digital Health Technologies: Scoping Review
Source: J Med Internet Res. 2024 Aug 28;26:e50251. doi: 10.2196/50251 (PMC11391152; doi:10.2196/50251)
Supplement: Multimedia Appendix 6 [file jmir_v26i1e50251_app6.docx]

Multimedia appendix 4

List of evaluation approaches

| **Evaluation_approach** | **Frequency** | **Percentage** |
| --- | --- | --- |
| RCT | 171 | 19.0% |
| Survey Research | 94 | 10.5% |
| Cohort study (prospective) | 75 | 8.3% |
| Interview study | 59 | 6.6% |
| Cohort study (retrospective) | 54 | 6.0% |
| Mixed method study design | 42 | 4.7% |
| Systematic review | 39 | 4.3% |
| Cross-sectional study | 36 | 4.0% |
| Feasibility study | 31 | 3.4% |
| Pilot study | 28 | 3.1% |
| Focus group study | 25 | 2.8% |
| Observational study | 20 | 2.2% |
| Pre–post study design | 15 | 1.7% |
| descriptive study | 14 | 1.6% |
| User-centered design (UDC) methods | 14 | 1.6% |
| Case series | 10 | 1.1% |
| Methods comparison study | 10 | 1.1% |
| Economic evaluation | 8 | 0.9% |
| Longitudinal studies | 8 | 0.9% |
| Simulation study | 6 | 0.7% |
| Before-and-after (BA) studies | 5 | 0.6% |
| Case–control studies | 5 | 0.6% |
| Cost-effectiveness analysis | 5 | 0.6% |
| Evaluative Questionnaire for E-health Tools (EQET) | 5 | 0.6% |
| Narrative review | 5 | 0.6% |
| comparative study | 4 | 0.4% |
| Participatory design | 4 | 0.4% |
| Retrospective analysis | 4 | 0.4% |
| Single-case experiment (N=1 trial) | 4 | 0.4% |
| Think aloud method | 4 | 0.4% |
| Practical clinical trial (PCT) | 3 | 0.3% |
| Scoping review | 3 | 0.3% |
| Big data analyse | 2 | 0.2% |
| Clinical trial | 2 | 0.2% |
| Crossover study | 2 | 0.2% |
| Exploratory Research | 2 | 0.2% |
| Heuristic evaluation | 2 | 0.2% |
| integrative review | 2 | 0.2% |
| Medical Research Council framework for complex interventions | 2 | 0.2% |
| Non-inferiority trial design | 2 | 0.2% |
| Open trial | 2 | 0.2% |
| Quasi-experimental study | 2 | 0.2% |
| Retrospective review | 2 | 0.2% |
| Technology Acceptance Model (TAM) | 2 | 0.2% |
| validation study | 2 | 0.2% |
| A test-retest design | 1 | 0.1% |
| Adaptive design | 1 | 0.1% |
| algorithm validation study | 1 | 0.1% |
| assessing sensitivity / specificity | 1 | 0.1% |
| CeHRes Roadmap | 1 | 0.1% |
| Coding Conversation Analysis | 1 | 0.1% |
| content analysis | 1 | 0.1% |
| Correlational study | 1 | 0.1% |
| Cross-sectional survey | 1 | 0.1% |
| decision analytic model-based health technology assessment | 1 | 0.1% |
| Delphi study | 1 | 0.1% |
| descriptive crossover design | 1 | 0.1% |
| descriptive qualitative assessment | 1 | 0.1% |
| Diagnostic accuracy evaluation study | 1 | 0.1% |
| Efficacy evaluation study | 1 | 0.1% |
| eHEALS | 1 | 0.1% |
| Ethnographic methods | 1 | 0.1% |
| ex vivo and in vivo study | 1 | 0.1% |
| Experimental study | 1 | 0.1% |
| Field testing | 1 | 0.1% |
| Fit between Individuals, Task and Technology (FITT) framework | 1 | 0.1% |
| Five-stage model for comprehensive research on telehealth | 1 | 0.1% |
| Fogg behavior model | 1 | 0.1% |
| Interrupted time series analysis | 1 | 0.1% |
| interview and Consolidated Framework for Implementation Research (CFIR) | 1 | 0.1% |
| Life-cycle–based approach | 1 | 0.1% |
| Logistic regression | 1 | 0.1% |
| Matched cohort study design | 1 | 0.1% |
| mHealth Development and Evaluation Framework | 1 | 0.1% |
| Micro-randomized trial | 1 | 0.1% |
| Mixed-effects regression | 1 | 0.1% |
| Model of Fog | 1 | 0.1% |
| Multiphase Optimization Strategy | 1 | 0.1% |
| Multiple case study design | 1 | 0.1% |
| multivariate analyses | 1 | 0.1% |
| Non-randomised controlled trial (NRCT) | 1 | 0.1% |
| Non-randomized group comparison study | 1 | 0.1% |
| Nonadoption, abandonment, scale-up, spread, and sustainability (NASSS) framework | 1 | 0.1% |
| Normalization process model | 1 | 0.1% |
| Patient Reported Outcome Measures (PROMs) | 1 | 0.1% |
| Pentagram model | 1 | 0.1% |
| pre- and post-intervention questionnaires | 1 | 0.1% |
| Preliminary report | 1 | 0.1% |
| proces evaluation | 1 | 0.1% |
| Prospective, single-group, open-label, siteless, pragmatic study | 1 | 0.1% |
| prospective, split-half, within-subjects design | 1 | 0.1% |
| Questionnaire for User Interface Satisfaction (QUIS) | 1 | 0.1% |
| RE-AIM framework (Reach, Effectiveness, Adoption, Implementation and Maintenance) | 1 | 0.1% |
| Repeated measures design | 1 | 0.1% |
| Retrospective record review | 1 | 0.1% |
| review and content analysis | 1 | 0.1% |
| Sequential Multiple Assignment Randomized Trial (SMART) | 1 | 0.1% |
| Statistical process control | 1 | 0.1% |
| Statististical analysis | 1 | 0.1% |
| study of diagnostic accuracy | 1 | 0.1% |
| system usability scale (SUS) questionnaire | 1 | 0.1% |
| systematic instructional design (SID). | 1 | 0.1% |
| Systems  development life-cycle (SDLC) methodology | 1 | 0.1% |
| Technology Readiness and Acceptance Model (TRAM) | 1 | 0.1% |
| The Multigroup Multilevel Categorical Latent Growth Curve Models | 1 | 0.1% |
| Usability testing: the 5E approach | 1 | 0.1% |
| Vignette study | 1 | 0.1% |
| Wait list control group design | 1 | 0.1% |
|  |  |  |
|  |  |  |
|  |  |  |
|  |  |  |
|  |  |  |
|  |  |  |
|  |  |  |
|  |  |  |
|  |  |  |
|  |  |  |
|  |  |  |
|  |  |  |
|  |  |  |
|  |  |  |
|  |  |  |
|  |  |  |
|  |  |  |
