# Supplementary material for: Approaches to Evaluating Digital Health Technologies: Scoping Review
Source: J Med Internet Res. 2024 Aug 28;26:e50251. doi: 10.2196/50251 (PMC11391152; doi:10.2196/50251)

primary_user vs functional_class


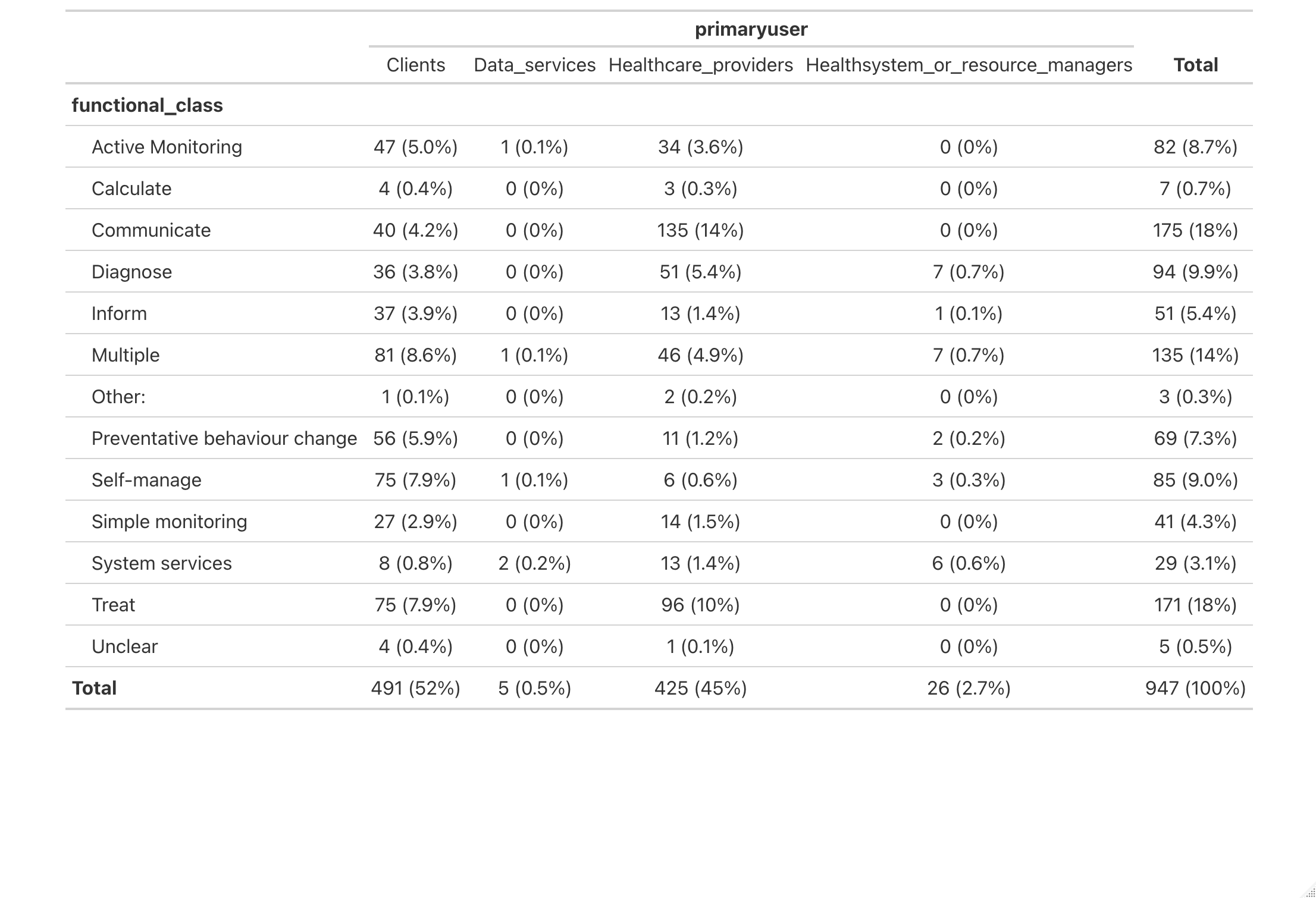


primary_user vs evaluation_study_phase


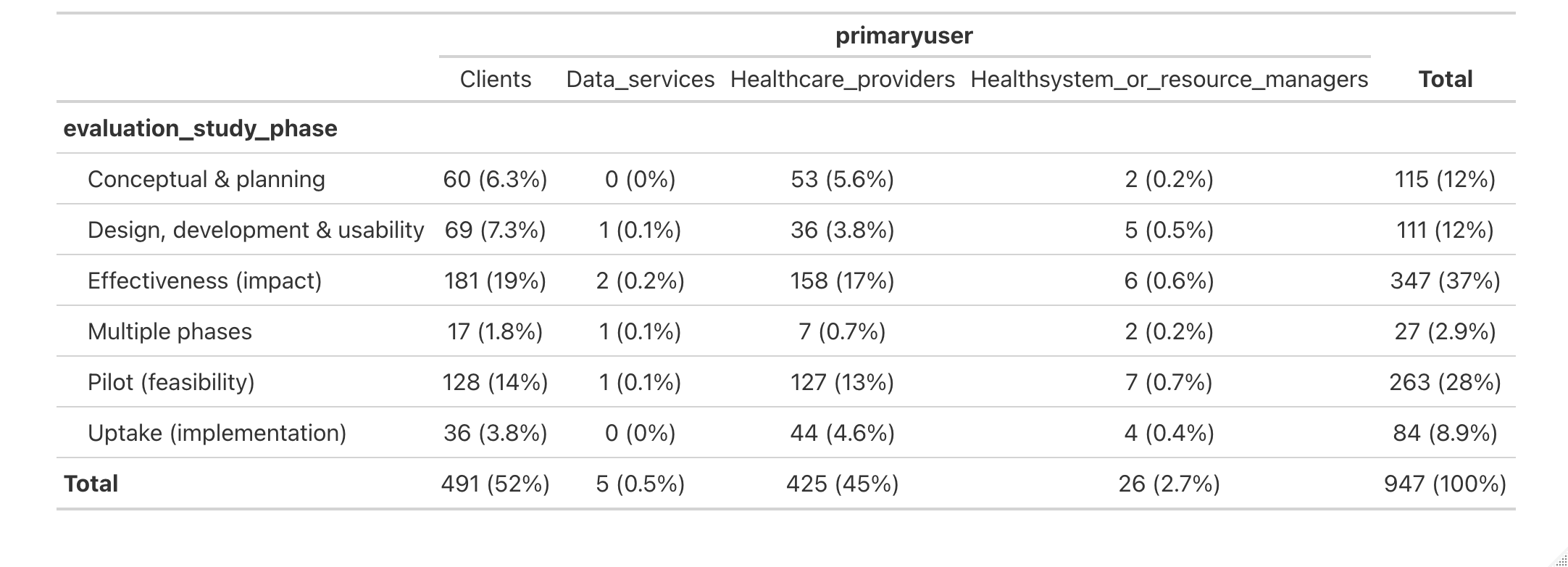


primary_user vs top 8 evaluation_approach


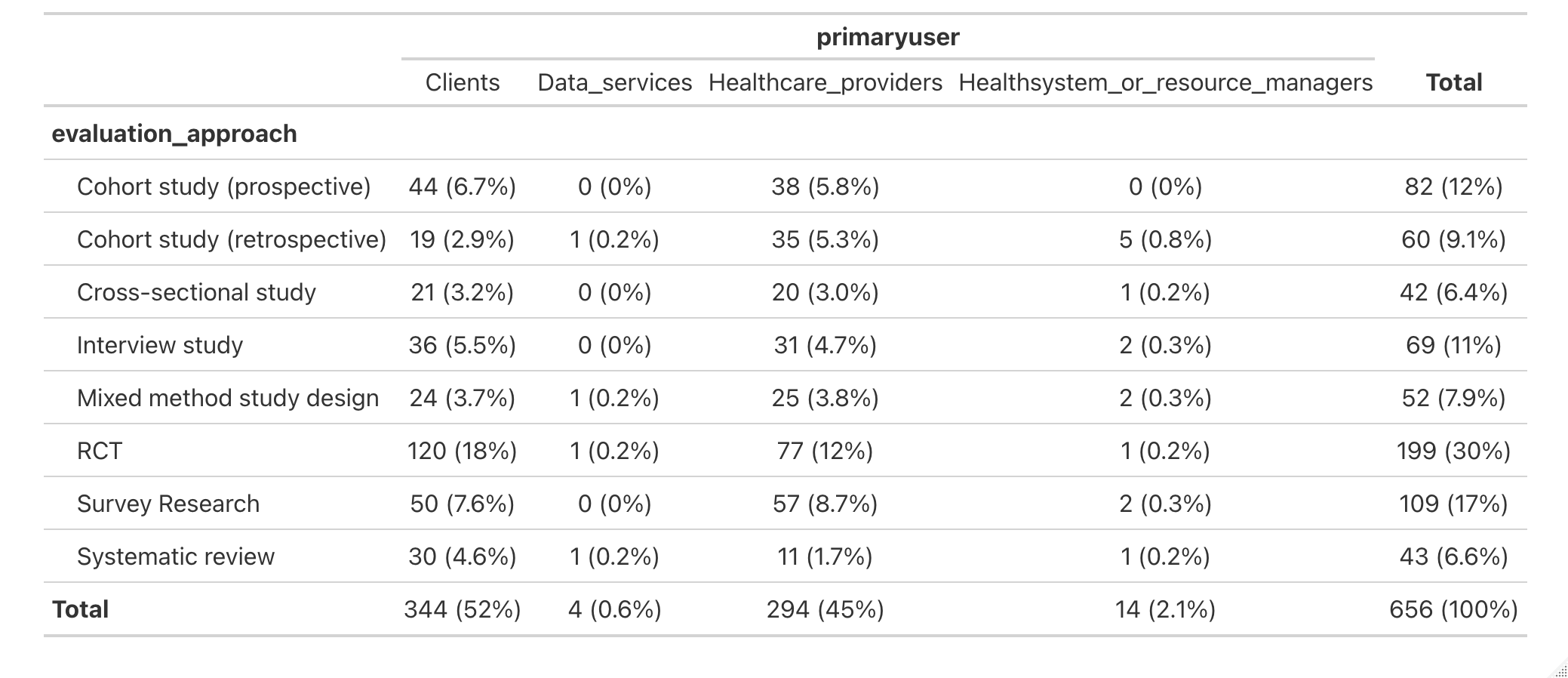


functional_class vs evalation_study_phase


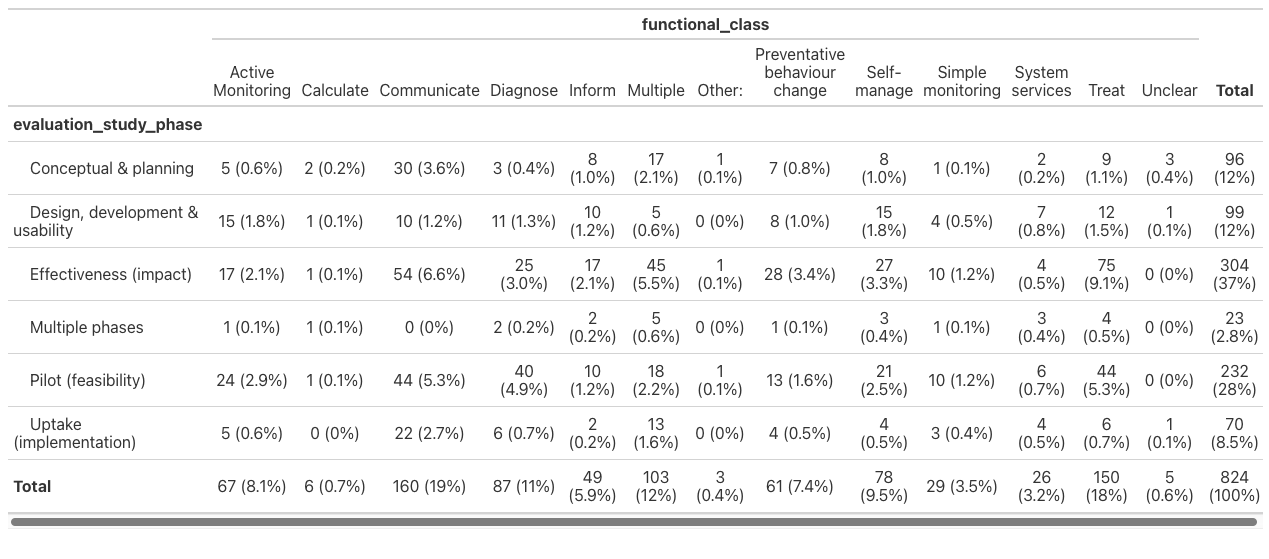


functional_class vs top 8 evaluation_approach


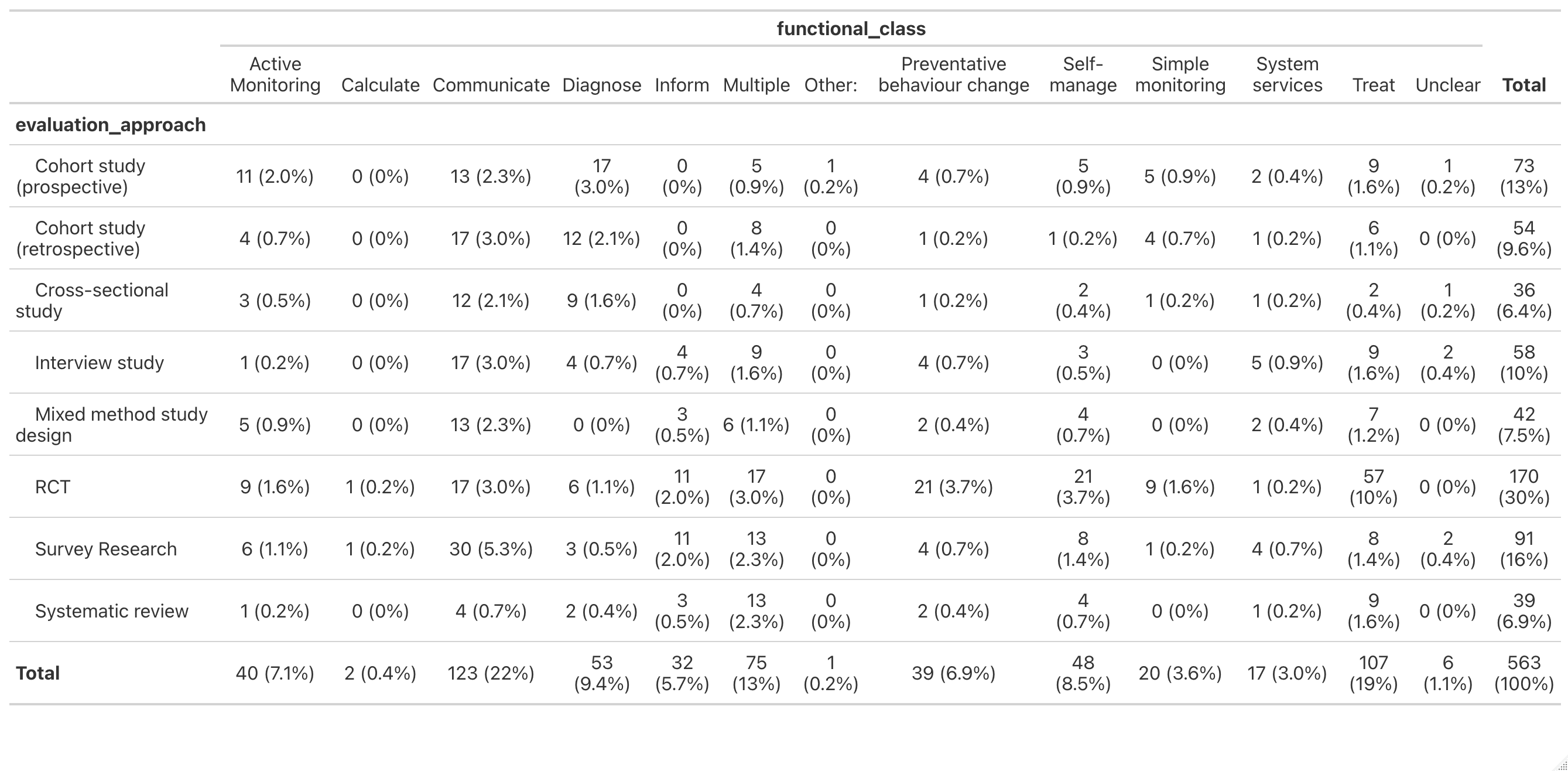


evaluation_study_phase vs top 5 medical_specialty


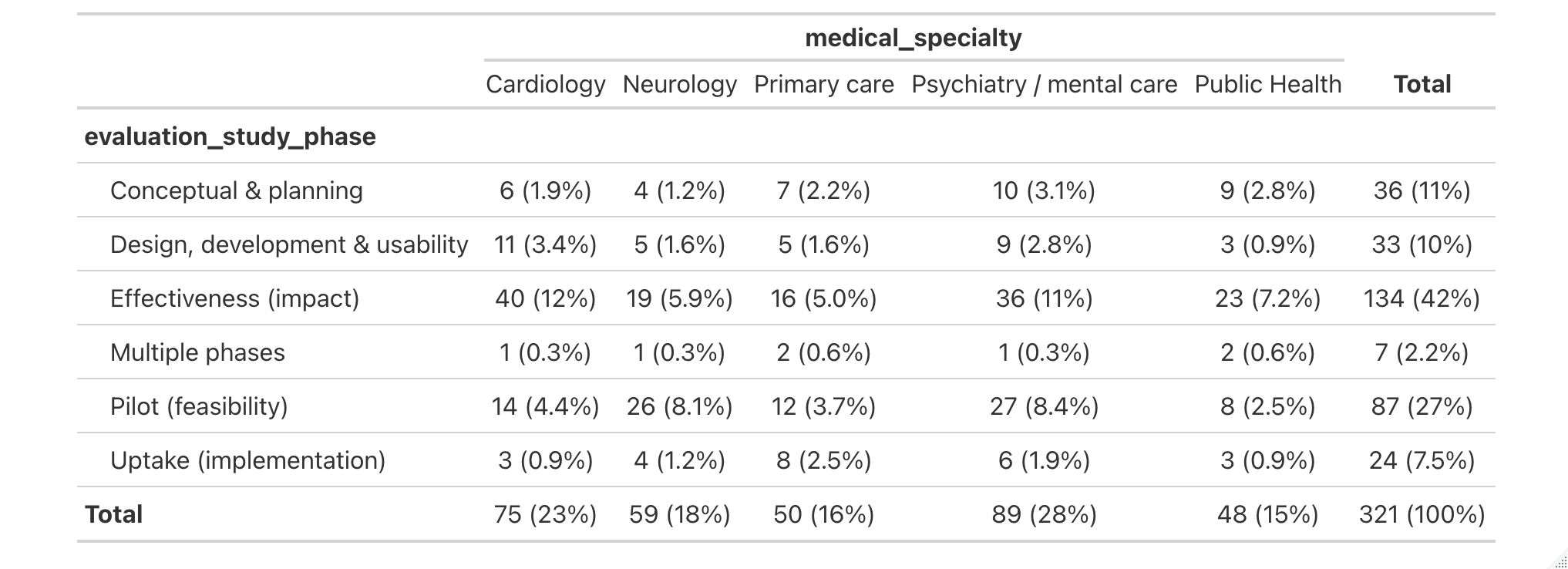


evaluation_study_phase vs top 8 evaluation_approach


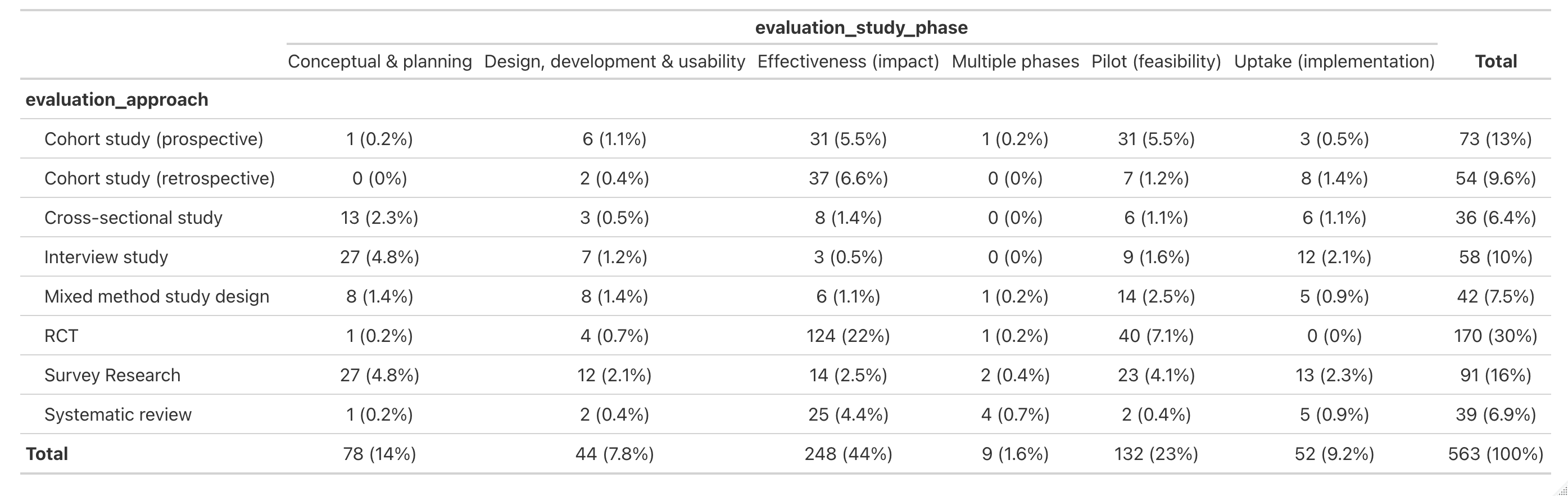


top 5 medical_specialty vs top 8 evaluation_approach


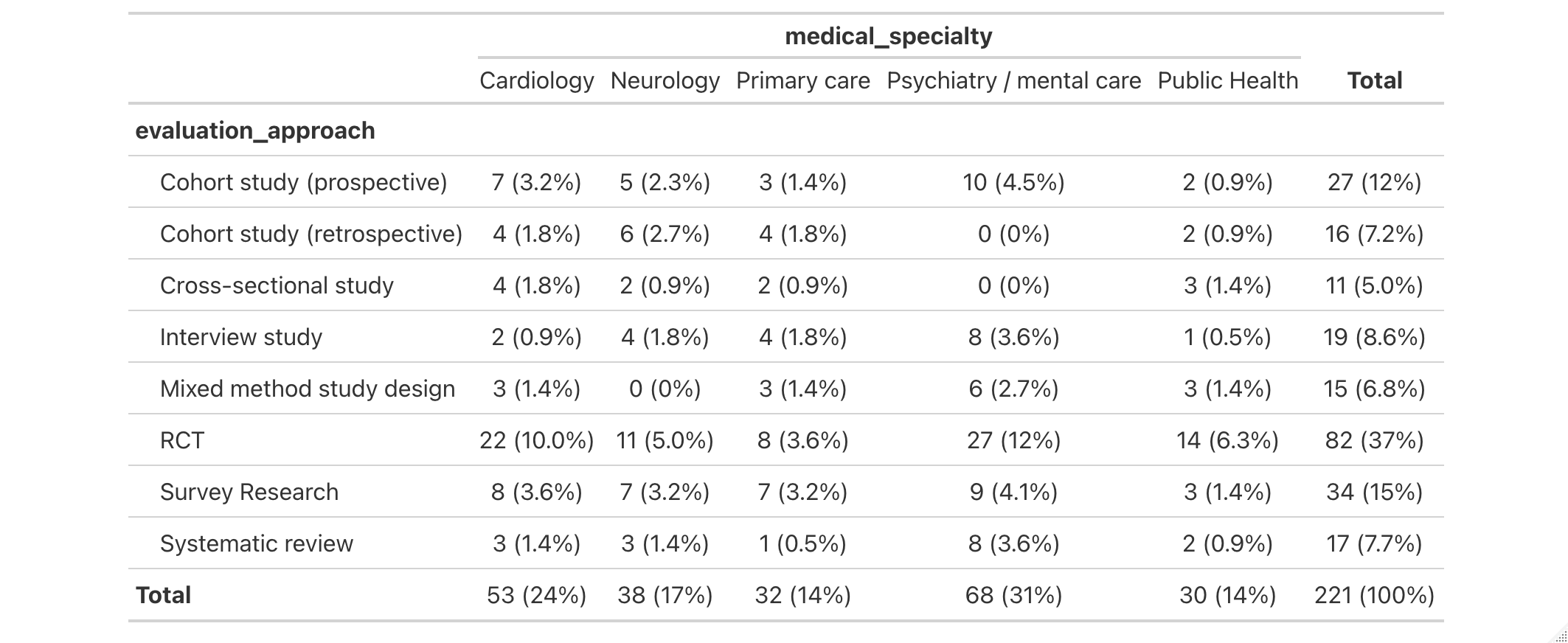

Supplement: Multimedia Appendix 7 [file jmir_v26i1e50251_app7.docx]
